# High Systolic Blood Pressure of High-Income African American Children

**DOI:** 10.1007/s40615-023-01668-5

**Published:** 2023-07-07

**Authors:** Shervin Assari, Babak Najand, Seyedeh Mohaddeseh Khatami

**Affiliations:** 1 Department of Family Medicine, Charles R Drew University of Medicine and Science, 1731 E 120Th St, Los Angeles, CA 90059 USA; 2 Department of Urban Public Health, Charles R Drew University of Medicine and Science, Los Angeles, CA USA; 3 School of Nursing, Charles R. Drew University of Medicine and Science, Los Angeles, CA USA; 4 Marginalization-Related Diminished Returns (MDRs), Los Angeles, CA USA; 5 Department of Internal Medicine, University of Toronto, Toronto, Ontario Canada

**Keywords:** Race, Socioeconomic position, Blood pressure, Body mass index

## Abstract

**Background:**

According to the Minorities’ Diminished Returns (MDRs) theory, racism may reduce the health returns of family socioeconomic status (SEP) resources such as family income for racial minorities, particularly African Americans, compared to Whites. However, we are unaware of any previous studies on racial variation in the protective effects of family income on children’s blood pressure.

**Aim:**

We conducted this study to compare the overall effects of family income on pre-adolescents’ systolic and diastolic blood pressure, test racial variation in this effect, and investigate whether racial variation in this regard is due to racial differences in body mass index.

**Methods:**

In this cross-sectional study, we analyzed data from 4007 racially diverse US children 9–10 years old. The independent variable was family income measured as a three-level categorical variable: less than $50 K USD, 50–100 K USD, and 100 + K USD. The primary outcomes were systolic and diastolic blood pressure measured up to three times at 1-min time intervals. Body mass index was the mediator. Mixed-effects regression models were used for data analysis to adjust for data nested to the centers, families, and individuals. Age, gender, parental education, family structure, and Latino ethnicity were covariates.

**Results:**

In the pooled sample and in the absence of interaction terms in the model, family income did not show an inverse association with children’s systolic (for 100 + K USD family income: *β* =  − 0.71, *p* = 0.233 and for 50–100 K USD family income: *β* = 0.01, *p* = 0.989) or diastolic blood pressure (for 100 + K USD family income: *β* =  − 0.66, *p* = 0.172 and for 50–100 K USD family income: *β* = 0.23, *p* = 0.600). However, race showed a significant interaction with family income on systolic blood pressure (for 50–100 K USD × African American: *β* = 2.75, *p* = 0.034), suggesting higher systolic blood pressure of African American adolescents from high-income backgrounds. Racial variation in the protective effect of family income on systolic BP was no more significant (for 50–100 K USD × African American: *β* = 2.14, *p* = 0.149) after we controlled for body mass index (BMI), which was higher for African American than White adolescents.

**Conclusion:**

The association between high family income and reduced systolic blood pressure in pre-adolescence might be weaker for African Americans compared to Whites, a difference that African American adolescents’ higher body mass index can explain.

## Introduction

Due to historical racism in the USA, race [1] and socioeconomic position (SEP) [2] closely overlap, as on average African Americans have lower SEP, such as family income, compared to Whites [3]. As race and low income correlate with health outcomes, it is essential to decompose the role of race and SEP on various health outcomes [3]. In addition, as low SEP, such as low income, may be one of the mechanisms that connect race to health and development [4], it is important to test the additive effects of race and family income [5, 6]. Some research has suggested that higher exposure to adverse life experiences might fully mediate the effects of SEP on health outcomes [7]. In addition, as the effects of SEP indicators such as family income may vary by race, it is important to test multiplicative (joint) effects of race and family income on health outcomes [3].

A recent body of cross-sectional literature has suggested that the protective effects of SEP on adverse life experiences differ for White and racial minority families, a finding that holds for adults [8–11] and adolescents [12, 13]. In a study that used the baseline of the Adolescent Brain Cognitive Development (ABCD) study, high family income and education were protective against adverse life experiences of 9–10-year-old pre-adolescents; however, these effects were weaker for African American than White pre-adolescents. The study did not include other racial groups, did not follow participants over time, and did not compare the protective effects of family structure by race [14]. Another cross-sectional study showed a high prevalence of spanking of high SEP-African American children [15]. In another cross-sectional study, highly educated African American adults reported a higher level of occupation-related adverse life experiences, while highly educated White adults reported a low number of negative life experiences due to occupation [16]. In a cross-sectional survey, African Americans with high human capital remained at higher risk of poverty than highly educated Whites [17, 18]. This is partly because African American families experience adversities across all SEP levels, while Whites experience less stress when their SEP is high [19]. Multiple reports also document high race-related discrimination against high SEP African American families [8–11]. Another study also showed that high-income African American families might remain in dangerous neighborhoods [20]. However, this research has never tested diminished returns of family income for childhood BP.

According to Minorities’ Diminished Returns (MDRs), relative to Whites, African Americans show weaker effects of family SEP indicators such as family income on tangible health outcomes [21, 22]. Various SEP indicators, such as income, tend to generate fewer health outcomes for the members of racial minority groups. Racial minority groups may not have the access, literacy, and connections to successfully navigate the available resource systems to secure tangible outcomes [22–27]. However, most of this literature is on African American families who show weaker effects of family income on various outcomes relative to Whites [21, 22, 25, 28, 29]. Thus, there is a need to include other racial groups in such an analysis.

High blood pressure (BP), or hypertension, is the most common non-communicable disease in the USA and many developed countries. High BP is a significant risk factor for serious health conditions such as stroke, heart disease, and dementia and thus requires attention. Recent research suggests that elevated BP in childhood may contribute to the development of high BP later in life, including adulthood. As there are distinct trajectories of BP from childhood to adulthood, early BP is an indicator of such trajectories that can provide valuable information about future BP and associated health outcomes. White race and family income are associated with lower BP, including childhood BP. However, compared to Whites, African Americans have higher BP. One can think of social (e.g., poverty) and biological (e.g., genetic) correlates of high blood pressure in children and adolescents. Regarding biological factors, in a longitudinal study, African American children, compared to White participants, demonstrated a larger increase in insulin resistance from childhood to adolescence [30]. On the other hand, many social determinants, such as family income, have been associated with lower childhood BP. Therefore, studying predictors of childhood BP is crucial to inform interventions to prevent hypertension in adulthood. In this context, there is a need for studies that have a large racially diverse sample that reflects US demographic distribution. The ABCD study has collected SEP, race, and BP data in a large cohort of pre-adolescents which makes it feasible to examine racial variations in the association between family income as one of the main SEP indicators and childhood BP during pre-adolescence.

## Aim

The study aimed to investigate the effect of family income as a main SEP indicator on childhood BP and whether such an effect shows any variation by race. We also tested if any racial difference in the effect of family income on BP is due to racial differences in body mass index (BMI). We built our study based on the MDRs literature, which previously documented weaker SES effects for African American than White pre-adolescents. Understanding the factors contributing to the development of variation in BP in childhood can inform public health interventions aimed at reducing the prevalence of hypertension in adulthood. Therefore, it is important to study childhood BP and its association with SEP indicators and race. The findings of this study can provide valuable information about the development of hypertension in childhood and the potential impact of social determinants on this process. Such data can inform public health programs and clinical interventions to prevent hypertension in adulthood, particularly for populations with increased risk.

## Methods

### Design and Settings

**Fig. 1:**
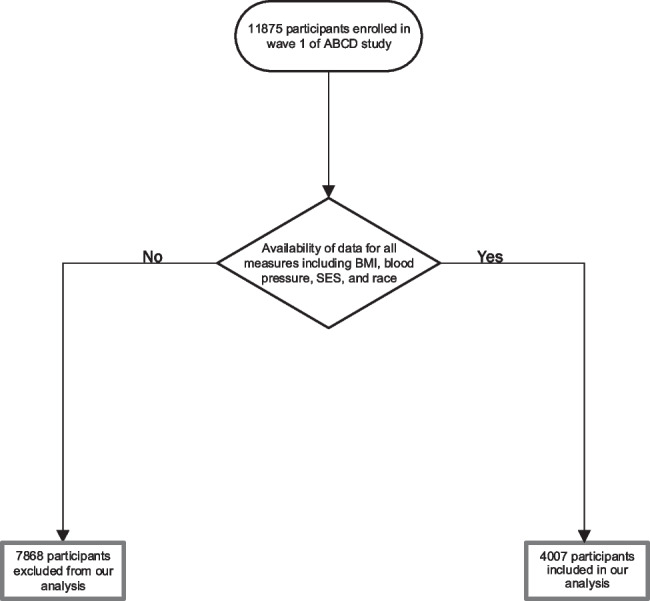
Overview of sample selection

This cross-sectional study was a secondary analysis of the existing data. Data came from the Adolescent Brain Cognitive Development (ABCD) study [31–35], a national longitudinal investigation of a diverse sample of children at pre-adolescence. More information about ABCD’s purpose, methodology, and measurement is available elsewhere [31, 36]. Some advantages of the ABCD data include a longitudinal, national, large, and diverse sample of race, SEP, and geography [31–35]. The ABCD sampling was predominantly from schools nested in cities across states [37]. For our secondary analysis, we included 9- and 10-year pre-adolescents who enrolled in the first wave of ABCD and whose data for any of our study’s variables (including family income, systolic and diastolic blood pressure, body mass index (BMI), race, age, gender, family structure, parental education, and ethnicity) were not missing (Fig. 1).

### Study Variables

The study variables included two demographic factors, namely age and gender, and three SEP indicators: family education, family income and family structure, race, ethnicity, BMI, and systolic and diastolic BP during pre-adolescence.

### Confounders

Age, gender, family structure, parental education, and ethnicity were the confounders. Parents reported the child’s date of birth, and the child’s age was calculated in the month at the time of baseline data collection. Age was treated as a continuous measure in months. Gender was a dichotomous variable, with males coded as 1 and females coded as 0. Parents reported, “What is the highest grade or level of school you have completed or the highest degree you have received?” They also reported the highest educational attainment of their partner. Responses in this study included a five-level categorical variable as below: less than high school, high school completed, some college, college graduated, and graduate studies. This variable captured both maternal and paternal education. The reference group was the lowest education, which was less than high school. Parents reported their marital status. Family structure was a dichotomous variable and coded 1 for married and 0 for any unmarried status. Latino ethnicity was reported by the parent.

### Independent Variable

#### Family Income

Family income was a three-level categorical measure. The exact question was, “What is your total combined family income for the past 12 months? This should include income (before taxes and deductions) from all sources, wages, rent from properties, social security, disability and veteran’s benefits, unemployment benefits, workman.” Responses included less than $50,000, between $50,000 and $100,000, and $100,000 or more.

### Dependent Variables

Systolic and diastolic blood pressures were measured at baseline. These were based on three measurements depicted in Box 1. The average of the three measures was used.

Box 1 Blood pressure measurements.

**Table:** 

First appointment	Systolic blood pressure 1st reading
	Diastolic blood pressure 1st reading
	Pulse rate 1st reading
Second appointment	Systolic blood pressure 2nd reading
	Diastolic blood pressure 2nd reading
	Pulse rate 2nd reading
Third appointment	Systolic blood pressure 3rd reading
	Diastolic blood pressure 3rd reading
	Pulse rate 3rd reading

### Mediator

#### Body Mass Index (BMI)

BMI was calculated based on measured height (in feet and inches) and weight (in pounds). The following formula was used to calculate BMI: BMI equals a person’s weight in kilograms divided by the square of height in meters.

### Moderator

#### Race

Race was identified as African American or Black, Asian, Mixed/Other, and White (reference category) reported by parents.

### Data Analysis

We used the Data Exploration and Analysis Portal (DEAP) platform for our data analysis. Average (standard deviation [SD]) and *n* = frequency (%) were described overall and by race. For multivariable analysis, we ran mixed-effects regression models that adjusted for nested data and multiple observations per individual, family, and center. All models were performed in the pooled sample that included all racial groups. While *model 1* did not include any interaction terms, *model 2* included interaction terms between race and family income. Both models controlled for age, gender, parental education, family structure, center and family. The outcome was childhood BP during pre-adolescence. Predictor was family income as a categorical outcome. The moderator was race. The outcomes were continuous variables (systolic or diastolic BP). Standardized beta coefficients (*b*), 95% CI, and *p* value were reported. Appendix 1 presents our formula for analysis in DEAP. Appendix 2 shows the distribution of our variables and regression error terms.

### Ethical Aspect

The ethics of the ABCD study protocol was approved by the University of California, San Diego (UCSD) Institutional Review Board (IRB). All adolescents provided assent, and all parents signed informed consent. For more information on the IRB and ethics of the ABCD study, please consult here [36]. For this analysis, we used fully de-identified data. Our study was deemed exempt from a full IRB review by the Charles R. Drew University of Medicine and Science.

## Results

### Descriptives

Table 1 presents descriptive statistics of the pooled sample and by race. The current analysis was performed on 4007 9–10-year-old children who were either White (*n* = 2621 (65.4%)), African American (*n* = 575 (14.3%)), Asian (*n* = 95 (2.4%)), and other/mixed race (*n* = 716 (17.9%)). Racial groups differed in parental education, family income, ethnicity, family structure, systolic and diastolic BP, and BMI during pre-adolescence (all *p* values are < 0.001).

**Table 1:** Socio-demographic data overall and by race

Vars	Level	Overall	White	African American	Asian	Other/mixed	*p*
***N***		4007	2621	575	95	716	
		Mean (SD)	Mean (SD)	Mean (SD)	Mean (SD)	Mean (SD)	
Age (month)*		143.55 (7.95)	143.63 (7.91)	143.74 (7.83)	143.54 (7.91)	143.07 (8.16)	0.361
Systolic blood pressure (BP)		102.34 (10.63)	101.88 (10.40)	104.88 (10.93)	100.53 (10.59)	102.23 (10.92)	< 0.001
Diastolic blood pressure (BP)		60.35 (8.71)	59.88 (8.59)	62.51 (9.05)	61.49 (8.66)	60.14 (8.63)	< 0.001
Body mass index (BMI)			19.79 (4.05)	22.36 (4.88)	20.16 (4.06)	20.81 (4.53)	< 0.001
		*n* (%)	*n* (%)	*n* (%)	*n* (%)	*n* (%)	
Married family*	No	1142 (28.5)	472 (18.0)	416 (72.3)	12 (12.6)	242 (33.8)	< 0.001
	Yes	2865 (71.5)	2149 (82.0)	159 (27.7)	83 (87.4)	474 (66.2)	
Sex	Female	1916 (47.8)	1240 (47.3)	290 (50.4)	43 (45.3)	343 (47.9)	0.552
	Male	2091 (52.2)	1381 (52.7)	285 (49.6)	52 (54.7)	373 (52.1)	
Family education*	< HS diploma	138 (3.4)	45 (1.7)	56 (9.7)	4 (4.2)	33 (4.6)	< 0.001
	HS diploma/GED	313 (7.8)	112 (4.3)	123 (21.4)	1 (1.1)	77 (10.8)	
	Some college	972 (24.3)	502 (19.2)	219 (38.1)	5 (5.3)	246 (34.4)	
	Bachelor	1102 (27.5)	817 (31.2)	87 (15.1)	24 (25.3)	174 (24.3)	
	Post graduate degree	1482 (37.0)	1145 (43.7)	90 (15.7)	61 (64.2)	186 (26.0)	
Family income*	< 50 K	1110 (27.7)	431 (16.4)	388 (67.5)	14 (14.7)	277 (38.7)	< 0.001
	≥ 50 K and < 100 K	1156 (28.8)	815 (31.1)	119 (20.7)	23 (24.2)	199 (27.8)	
	≥ 100 K	1741 (43.4)	1375 (52.5)	68 (11.8)	58 (61.1)	240 (33.5)	
Hispanic	No	3288 (82.1)	2228 (85.0)	545 (94.8)	87 (91.6)	428 (59.8)	< 0.001
	Yes	719 (17.9)	393 (15.0)	30 (5.2)	8 (8.4)	288 (40.2)	

*p < 0.05 for comparison of racial groups

### Multivariate Analysis

Table 2 shows the effect sizes, and the model fits. Models with interactions showed a better fit (for systolic blood pressure: model 2 (with interactions) has Δ*R*-squared% = 0.82% versus model 1 (main effects) has *ΔR*-squared% = 0.07%; for diastolic blood pressure: model 2 (with interaction) has Δ*R*-squared% = 0.88% versus model 1 (main effects) has Δ*R*-squared% = 0.16%). However, the models with BMI had smaller sample sizes, so their fits were lower than other models.

**Table 2:** Fit across models without and with interactions and with BMI as a mediator

		Model 1 Main effects	Model 2 With interaction	Model 3 With BMI
*N*	Systolic	4007	4007	2915
*R*-squared		0.0382	0.04064	0.11841
Δ*R*-squared		0.00071	0.00817	0.0042
Δ*R*-squared%		0.07%	0.82%	0.42%
*N*	Diastolic	4007	4007	2915
*R*-squared		0.01689	0.01946	0.07164
Δ*R*-squared		0.00155	0.00879	0.00595
Δ*R*-squared%		0.16%	0.88%	0.6%

### Systolic BP

Table 3 summarizes the results of three regression models in the overall (pooled) sample with systolic BP as the outcome. *Model 1* (main effect model) showed an association between race (African American: *β* = 2.46, *p* = 0.000) but not family income (all *p* values > 0.05) with systolic BP during pre-adolescence. Model 2 showed an interaction between race and family income (for 50–100 K USD × African American: *β* = 2.75, *p* = 0.034), suggesting that the association between family income with systolic BP during pre-adolescence varied by race (weaker for African American than White adolescents). These racial differences in the association between family income and systolic BP were due to BMI, as shown by model 3 (for 50–100 K USD × African American: *β* = 2.14, *p* = 0.149).

**Table 3:** Summary of regressions of systolic blood pressure without (model 1) and with interactions between race and family income (model 2) and with BMI as a mediator (model 3)

	Estimate	SE	Pr( >|*t*|)	
Model 1				
Family education				
Less than HS diploma				
HS diploma/GED	0.33	1.09	0.765	
Some college	− 0.48	1.00	0.631	
Bachelor	− 1.26	1.05	0.231	
Post graduate degree	− 1.14	1.06	0.285	
Age	0.19	0.02	< 1e-6	***
Sex (male)	0.90	0.33	0.006	**
Race				
White				
African American	2.46	0.59	0.000	***
Asian	− 0.84	1.13	0.460	
Other/mixed	0.51	0.48	0.288	
Married	− 0.47	0.46	0.314	
Family income				
< 50 K				
≥ 100 K	− 0.71	0.59	0.233	
≥ 50 K and < 100 K	0.01	0.53	0.989	
Hispanic	0.30	0.52	0.564	
Model 2				
Family income				
< 50 K				
≥ 100 K	− 0.75	0.70	0.285	
≥ 50 K and < 100 K	− 0.13	0.68	0.848	
Age	0.18	0.02	< 1e-6	***
Sex (male)	0.89	0.33	0.007	**
Family education				
< HS diploma				
HS diploma/GED	0.39	1.09	0.718	
Some college	− 0.56	1.00	0.577	
Bachelor	− 1.34	1.06	0.203	
Post graduate degree	− 1.21	1.07	0.257	
Race				
White				
African American	1.60	0.81	0.048	*
Asian	3.23	2.87	0.260	
Other/mixed	1.04	0.83	0.210	
Married family	− 0.52	0.46	0.261	
Hispanic	0.21	0.53	0.683	
Family income ≥ 100 K × African American	1.51	1.55	0.330	
Family income ≥ 50 K and < 100 K × African American	2.75	1.30	0.034	*
Family income ≥ 100 K × Asian	− 4.87	3.19	0.126	
Family income ≥ 50 K and < 100 K × Asian	− 4.56	3.60	0.205	
Family income ≥ 100 K × Other/mixed	− 0.63	1.11	0.571	
Family income ≥ 50 K and < 100 K × Other/mixed	− 1.16	1.17	0.325	
Model 3				
Family income				
< 50 K				
≥ 100 K	− 0.44	0.76	0.563	
≥ 50 K and < 100 K	0.19	0.74	0.796	
Age	0.18	0.02	< 1e-6	***
Sex (male)	1.17	0.36	0.001	**
Family education				
< HS diploma				
HS diploma/GED	0.43	1.24	0.731	
Some college	− 0.82	1.14	0.470	
Bachelor	− 0.68	1.19	0.571	
Post graduate degree	− 0.26	1.21	0.829	
Race				
White				
African American	0.60	0.92	0.514	
Asian	0.75	3.07	0.807	
Other/mixed	0.50	0.91	0.581	
Married family	− 0.18	0.51	0.718	
Hispanic	− 0.46	0.59	0.436	
Family income ≥ 100 K × African American	1.89	1.67	0.257	
Family income ≥ 50 K and < 100 K × African American	2.14	1.48	0.149	
Family income ≥ 100 K × Asian	− 2.02	3.38	0.550	
Family income ≥ 50 K and < 100 K × Asian	− 2.27	3.81	0.552	
Family income ≥ 100 K × Other/mixed	− 0.70	1.23	0.569	
Family income ≥ 50 K and < 100 K × Other/mixed	− 0.41	1.32	0.755	
BMI	0.69	0.04	< 1e-6	***

.*p* < .01; *p < .05; **p < .001; ***p < .00001

### Diastolic BP

Table 4 summarizes the results of three regression models in the overall (pooled) sample for diastolic BP as the outcome. Model 1 (main effect model) showed an association between race (African American: *β* = 1.80, *p* = 0.000) but not family income (all *p* values > 0.05) with diastolic BP during pre-adolescence. Model 2 (with interactions) did not show any interaction between African American race and family income (*p* value > 0.05). However, model 2 showed an interaction between Asian race and family income (for 50–100 K USD × Asian: β =  − 5.98, *p* = 0.042). BMI was a predictor of diastolic BP (*β* = 0.49, *p* < 0.00001), as shown by model 3.

**Table 4:** Summary of regressions of diastolic blood pressure without (model 1) and with interactions between race and family income (model 2) and with BMI as the mediator (model 3)

	Estimate	SE	Pr( >|*t*|)	
Model 1				
Family education				
Less than HS diploma				
HS diploma/GED	0.17	0.89	0.846	
Some college	− 0.47	0.82	0.568	
Bachelor	− 1.37	0.86	0.111	
Post graduate degree	− 1.04	0.87	0.232	
Age	0.03	0.02	0.078	
Sex (male)	− 0.64	0.27	0.018	*
Race				
White				
African American	1.80	0.48	0.000	***
Asian	1.70	0.93	0.067	
Other/mixed	0.06	0.39	0.876	
Married	0.00	0.38	0.994	
Family income				
< 50 K				
≥ 100 K	− 0.66	0.48	0.172	
≥ 50 K and < 100 K	0.23	0.44	0.600	
Hispanic	0.32	0.43	0.453	
Model 2				
Family income				
< 50 K				
≥ 100 K	− 0.57	0.57	0.324	
≥ 50 K and < 100 K	0.60	0.56	0.280	
Age	0.03	0.02	0.078	
Sex (male)	− 0.64	0.27	0.017	*
Family education				
< HS diploma				
HS diploma/GED	0.25	0.89	0.781	
Some college	− 0.45	0.82	0.583	
Bachelor	− 1.38	0.86	0.109	
Post graduate degree	− 1.03	0.87	0.238	
Race				
White				
African American	1.71	0.66	0.010	*
Asian	5.53	2.35	0.018	*
Other/mixed	0.50	0.67	0.458	
Married family	− 0.05	0.38	0.893	
Hispanic	0.38	0.43	0.372	
Family income ≥ 100 K × African American	0.03	1.26	0.979	
Family income ≥ 50 K and < 100 K × African American	0.69	1.06	0.516	
Family income ≥ 100 K × Asian	− 3.82	2.61	0.143	
Family income ≥ 50 K and < 100 K × Asian	− 5.98	2.95	0.042	*
Family income ≥ 100 K × Other/Mixed	0.18	0.90	0.844	
Family income ≥ 50 K and < 100 K × Other/mixed	− 1.78	0.96	0.063	
Model 3				
Family income				
< 50 K				
≥ 100 K	− 0.08	0.62	0.898	
≥ 50 K and < 100 K	0.87	0.61	0.153	
Age	0.01	0.02	0.656	
Sex (male)	− 0.34	0.30	0.253	
Family education				
< HS diploma				
HS diploma/GED	0.51	1.02	0.618	
Some college	− 0.04	0.93	0.968	
Bachelor	− 0.16	0.98	0.871	
Post graduate degree	− 0.01	0.99	0.991	
Race				
White				
African American	0.65	0.75	0.389	
Asian	6.32	2.51	0.012	*
Other/mixed	− 0.39	0.74	0.601	
Married family	0.29	0.41	0.486	
Hispanic	− 0.34	0.49	0.487	
Family income ≥ 100 K × African American	0.41	1.36	0.764	
Family income ≥ 50 K and < 100 K × African American	− 0.49	1.21	0.682	
Family income ≥ 100 K × Asian	− 4.66	2.77	0.093	
Family income ≥ 50 K and < 100 K × Asian	− 6.45	3.13	0.039	*
Family income ≥ 100 K × Other/mixed	1.19	1.00	0.235	
Family income ≥ 50 K and < 100 K × Other/mixed	− 1.21	1.08	0.260	
BMI	0.49	0.04	< 1e-6	***

*p* < 0.01; *p < 0.05; **p < 0.001; ***p < 0.00001

**Fig. 2:**
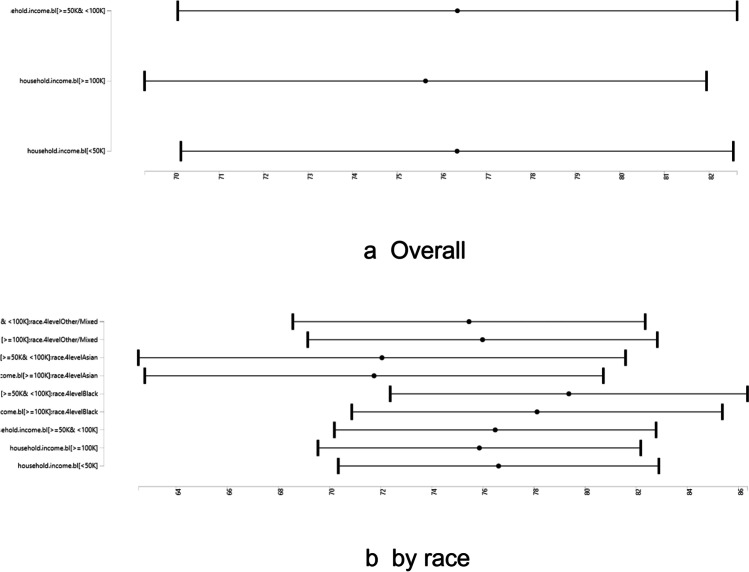
Overall and racial variation in the effect of income on childhood blood pressure during pre-adolescents

Figure 2 shows overall and racial variation in the association between family income and systolic BP.

## Discussion

We aimed to test the effects of family income on childhood BP during pre-adolescence. In the overall sample, we expected a protective effect of high family income on childhood BP during pre-adolescence. We also aimed to test the variation of such effect across diverse racial groups compared to White children. Our hypothesis was that BP might remain high in high-income racial minority children, particularly African American children. Such MDRs could help us understand why chronic conditions and health problems remain higher than expected in high SEP racial minority youth and adults [21, 22]. Our final aim was to test if the body mass index explains differential returns of high family income on BP by race.

Our finding is a replication and expansion of our previous observation. Our finding indicated diminished returns of family income on blood pressure during pre-adolescence for African American adolescents relative to White adolescents. In other words, a high income better translates to lower BP during adolescence for Whites than African American adolescents. Thus, while White children from high-income families remain protected against high BP and BMI during pre-adolescence, African American adolescents from high-income families experience high BP and BMI. While a large body of research has previously shown MDRs for other outcomes, the unique contribution of this study includes moving beyond the comparison of African Americans and Whites and testing MDRs of income on childhood BP for the first time. Using a cross-sectional design, we could test the association of family income on childhood BP during pre-adolescence.

Although high family income correlated with lower BP, this effect was weaker for African American than White adolescents. We explain this finding with an emerging literature that shows high depression [38], anxiety [26], stress [14, 39], poor diet [40], and higher body mass index [29, 41–43] in high SEP African American children and adolescents. There was also a report on the high BP of Latinos with high SEP, but we are unaware of any previous study on the high BP of African American adolescents with high SEP.

These results replicated and extended the MDRs. Similar MDRs are documented for various SEP resources, age groups, health or developmental outcomes, and marginalizing identities [21, 22]. Similar MDRs are shown for parental education, marital status, subjective SES, and household income [24, 41, 44, 45]. These remaining health risks may be one of the mechanisms for the trans-generational transmission of health inequalities in African American families. It is unknown what policies and conditions can break such negative cycles.

This paper suggests that clinicians should expect higher-than-expected BP and BMI in childhood who are in their pre-adolescence, similar to the previous research on higher-than-expected stress, obesity, anxiety, depression, suicide, and health problems in African American children [24, 41, 44, 45] and adults [10, 11, 46–48] from high SEP backgrounds. This result is significant because blood pressure and obesity contribute to many poor health outcomes [49].

Several pathophysiological mechanisms have been postulated for the repeatedly shown association between high BMI and hypertension [50–52]. Obesity demonstrates signs of adrenergic overactivity, including increased resting heart rate and serum norepinephrine levels [53]. Arterial baroreceptors, which play a central role in cardiovascular homeostasis, specifically the sympathetic tone, have been shown to be impaired in obesity-related hypertension [53]. In addition, the increased sympathetic outflow to the kidney results in the accelerated renal reabsorption of sodium [54] and activation of the renin-angiotensin system [55, 56]. A decrease in adiponectin, as a product of adipose tissue, has also been implicated in obesity-related hypertension through an endothelial-dependent pathway [57, 58]. Finally, insulin resistance seen in obesity [59] can lead to compensatory hyperinsulinemia, which in turn can result in increased salt reabsorption from the proximal tubule mediated by insulin receptor substrate 2 [60], whose function is preserved in insulin resistance [61].

Many explanations can be given to explain our findings. One is residential segregation which may reduce the returns of SEP for African American families. African American families have a higher tendency to remain in poor neighborhoods and attend worse schools across SEP lines [62, 63]. As a result of staying in a high-risk environment and social context, families of color and adolescents from high SEP backgrounds may remain at risk of environmental exposures to risk factors. In such a high-risk and low-resource social context, adolescents may be exposed to high-risk peers, aggression, danger, and other stressors [64, 65].

While MDRs are well described, the societal and contextual processes that explain MDRs are still unknown. Some researchers have attributed MDRs to structural and institutional racism [21, 66]. Childhood poverty may also be a mechanism that reduces later returns of SEP when the individual is an adult [67]. Prejudice and discrimination may interfere with the expected benefits of education, employment, income, and marriage for minority families [11, 47, 48]. Multilevel economic and societal mechanisms may carry the effects of MDRs across generations [21, 66].

More studies are needed on the role of peers, neighborhoods, schools, and family contexts in explaining the sustained risk in the lives of high-income African American adolescents. For example, it is unknown to what degree schools, neighborhoods, families, or peers explain residual adverse live conditions of high-income African Americans and to what degree such additional exposures translate to behavioral, cognitive, or emotional risk for adolescents [68]. Also, while policy can manipulate social environments, the remaining question is to what degree and what policies can equalize the returns of SEP indicators and what are the best solutions to reduce the harmful effects of segregation in the lives of high-income African American adolescents [69].

High blood pressure and BMI, closely linked to poor health, is a barrier against upward social mobility and healthy development. These adverse life experiences in the lives of minorities across class lines may reflect why we observe the intergenerational transition of adversities [70] and why upward social mobility is less common for racial minorities [71].

## Implications

Our findings suggest the following implications to design and implement policies and programs to reduce the harmful effects of high blood pressure and body mass index of high-income African American adolescents: first is to focus on reducing racism that reduces the returns of income on health and well-being of African American families and their children. Racism is the key mechanism that explains why family socioeconomic position does not generate the same health outcomes for racial minorities, particularly African Americans. Therefore, it is important to reduce anti-African American racism and discrimination in the USA. Policies should promote equity and inclusion in all aspects of life, such as education, employment, policing, and banking. The second is to increase BP, BMI measurement, and hypertension screening for African American adolescents, youth, and young adults. Screening may result in early detection of hypertension in African American populations. Such prevention and screening should not be limited to low-income families, as high-income families are also at risk of hypertension. Programs should also increase knowledge of African American communities about the blood pressure measurement of young adults. We need to increase the literacy of African American community members regarding the risks that remain high in middle-class communities of color. Such programs can leverage community involvement and family engagement to promote health in middle-class communities of color. The third is to reduce obesity and body mass index associated with high blood pressure in adolescents and adults. Programs that help with healthy nutrition and higher physical activities in African American communities and schools may help reduce the blood pressure of youth in such settings. Last but not least, address structural inequalities: structural inequalities, such as housing segregation and disparities in residential conditions, can contribute to stress, reduced access to healthy food options, and increased blood pressure for racial minorities across income levels. Policies should address these structural inequalities through initiatives that promote equity and reduce disparities in access to resources and opportunities.

## Limitations

This study had a few methodological limitations. First, our study was cross-sectional, so we cannot infer causal effects. We only focused on race as a marginalizing identity. Other marginalizing identities such as ethnicity, religion, sexual orientation, nativity, citizenship, and gender identity may also marginalize adolescents and their families, reducing the returns of their family SEP indicators [44, 72–74]. Similarly, this study only investigated family-level SEP indicators, some as a control variable and one as an independent variable. It is still being determined if neighborhood-level SEP indicators such as neighborhood poverty would also show any diminished returns. In this study, we used raw scores of BMI as an index for weight. Future studies can use further anthropometric indices such as weight-for-age, weight-for-height, height-for-age, and BMI-for-age, which the World Health Organization suggests for children and adolescents. In addition, we did not have access to other SEP indicators, such as wealth, home ownership, or family debt. Future research may also test neighborhood SEP and family wealth across broader groups with marginalized identities. Finally, by including contextual data from neighborhoods, schools, friends, and families, mechanisms of diminished returns of family income for racial minorities could be better understood.

## Conclusion

Compared to Whites, African American children from high-income backgrounds have higher BP during adolescence. High BP during the transition to adolescence in African American adolescents with high income may be due to their high body mass index. This is alarming and suggests that high-income African American children remain at a health risk that is disproportionate to their SEP. This is opposite to the low BP of White adolescents from high-income families. As childhood blood pressure and body mass index are predictors of hypertension and associated health problems later in life and development, more efforts should be made to prevent stress, increase physical activity, and improve diet in the lives of middle-class African American families.

## Data Availability

The ABCD data are available at the NIH NDA Website: https://nda.nih.gov/abcd/.

## Appendix 1 Model formula for DEAP

**Table:**

**Systolic** **Model 1** biospec_blood_pressure_sys_mean ~ household.income.bl + age + sex + race.4level + hisp + high.educ.bl + married.bl Random: ~ (1|abcd_site/rel_family_id) **Model 2** biospec_blood_pressure_sys_mean ~ household.income.bl + age + sex + race.4level + hisp + high.educ.bl + married.bl + household.income.bl * race.4level Random: ~ (1|abcd_site/rel_family_id) **Model 3** biospec_blood_pressure_sys_mean ~ household.income.bl + age + sex + race.4level + hisp + high.educ.bl + married.bl + anthro_bmi_calc + household.income.bl * race.4level Random: ~ (1|abcd_site/rel_family_id)	**Diastolic** **Model 1** biospec_blood_pressure_dia_mean ~ household.income.bl + age + sex + race.4level + hisp + high.educ.bl + married.bl Random: ~ (1|abcd_site/rel_family_id) **Model 2** biospec_blood_pressure_dia_mean ~ household.income.bl + age + sex + race.4level + hisp + high.educ.bl + married.bl + household.income.bl * race.4level Random: ~ (1|abcd_site/rel_family_id) **Model 3** biospec_blood_pressure_dia_mean ~ household.income.bl + age + sex + race.4level + hisp + high.educ.bl + married.bl + anthro_bmi_calc + household.income.bl * race.4level Random: ~ (1|abcd_site/rel_family_id)

## Code Availability

Codes are available in the Appendix.
